# Whole‐Genome Data to Investigate Recent and Historical Dog Introgression Patterns in Italian Wolves

**DOI:** 10.1002/ece3.72508

**Published:** 2025-11-27

**Authors:** D. Battilani, J. Ramos Madrigal, L. M. Hennelly, S. Gopalakrishnan, C. Vernesi, F. Mattucci, E. Fabbri, P. Ciucci, R. Caniglia

**Affiliations:** ^1^ Research and Innovation Centre, Fondazione Edmund Mach San Michele All'adige Italy; ^2^ Department of Biology and Biotechnologies “Charles Darwin” Università di Roma La Sapienza Roma Italy; ^3^ Area per la Genetica Della Conservazione, ISPRA Ozzano dell'Emilia Italy; ^4^ Center for Evolutionary Hologenomics The Globe Institute, University of Copenhagen Copenhagen Denmark; ^5^ Department of Biosciences Rice University Houston Texas USA; ^6^ Center for Conservation Genomics, Smithsonian's National zoo and Conservation Biology Institute Washington DC USA; ^7^ Institute of Biosciensces and Bioresources, Consiglio Nazionale Delle Ricerche Sesto Fiorentino Italy

**Keywords:** admixture timing, anthropogenic hybridization, behavior‐related genes, *Canis lupus italicus*, dog introgression, selection

## Abstract

Introgression resulting from anthropogenic hybridization may shape phenotypic traits in wild taxa, potentially altering species' ecology and human‐wildlife interactions. This is particularly relevant in large carnivores such as gray wolves ( *Canis lupus* ) that are expanding into human‐dominated landscapes. A notable example is the Italian wolf (*C. l. italicus*), which, after recovering from near extinction, now faces locally high levels of hybridization with the domestic dog (*C. l. familiaris*). Although wolf‐dog hybridization is known to affect morphology, its effect on other phenotypic traits remains poorly investigated. We analyzed worldwide‐distributed wolf and dog whole‐genome data to assess the extent and timing of dog ancestry in a sample of 17 Italian wolves, and to explore dog introgression at behavior‐related genes. Five Italian wolves, exhibiting varying levels of genome‐wide dog ancestry (1%–20%), were estimated to result from admixture events that occurred at least 3–7 generations before sampling (2005–2012). No overrepresentation of recent dog introgressions in behavior‐related genes of admixed Italian wolves was detected. However, we identified signals of historical dog introgressions under putative selection, involving genes linked to neuronal plasticity, photoreceptor development, and immune responses. One of such introgressions likely occurred ~4500 years ago during the Bronze Age and the other one ~1000 years ago during the Middle Ages, suggesting that historical admixture might have shaped the Italian wolf evolutionary trajectories. Although preliminary and warranting further analyzes, our results highlight the potential for phenotypic effects of wolf‐dog hybridization to persist across generations, showing that even limited non‐native ancestry can leave significant genomic footprints in wild populations.

## Graphical abstract

We analyzed whole‐genome data from Italian wolves and dogs to assess the extent, timing, and functional relevance of dog introgression in the Italian wolf. Five wolves showed recent admixture within the last 3–7 generations, while historical introgressions under selection dated to the Bronze and Middle Ages involved genes linked to neuronal plasticity, vision, and immune response. These findings suggest that even limited dog ancestry may leave lasting genomic and phenotypic imprints on wild wolf populations.

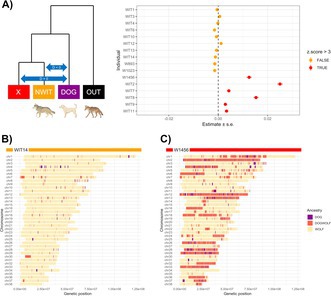

## 1 Introduction

Hybridization can play a pivotal role in shaping evolutionary processes (Todesco et al. 2016; Taylor and Larson 2019; Moran et al. 2021; Porretta and Canestrelli 2023). While natural hybridization typically unfolds over extended evolutionary timescales, anthropogenic hybridization, driven by human‐induced changes can occur rapidly (McFarlane and Pemberton 2019; Ottenburghs 2021). Anthropogenic hybridization may lead to genetic swamping, where introgression of non‐native ancestry compromises the gene pool of wild populations (Howard‐McCombe et al. 2023; Nussberger et al. 2023), or sometimes to adaptive introgression, potentially enhancing fitness through the acquisition of beneficial alleles (Mary et al. 2022; Fabbri et al. 2023; Münger et al. 2024). Ultimately, anthropogenic hybridization can alter the evolutionary trajectories of species, prompting conservation and management responses (Allendorf et al. 2001; Bohling 2016; Quilodrán et al. 2020).

A notable example of anthropogenic hybridization involves the gray wolf ( *Canis lupus* ) and its domestic counterpart, the dog ( *Canis lupus familiaris* ). Domesticated over 30,000 years ago (vonHoldt et al. 2010; Thalmann et al. 2013; Bergström et al. 2022), dogs were initially selected for traits aiding human tasks (e.g., hunting, herding, guarding) (Coppinger and Coppinger 2002), with a later shift toward aesthetics and companionship during and after the Victorian Age (Olmert 2018). Dogs and wolves can interbreed, and their fertile hybrids can backcross with both parental lineages, enabling the introgression of dog ancestry (i.e., dog introgression) into wolf populations (Vilà and Wayne 1999). While historical wolf‐dog hybridization (i.e., WDH) events likely occurred over thousands of years (Ciucani et al. 2023; Lobo et al. 2023), recent WDH has been genetically detected since the late 20th century (Vilà and Wayne 1999) across several European wolf populations, raising conservation concerns (Hindrikson et al. 2017; Salvatori et al. 2020).

In Europe, Italy has long been recognized as a hotspot of WDH (Boitani 1983; Boitani and Ciucci 1993; Ciucci and Boitani 1998). The Italian wolf (*Canis lupus italicus*) was on the brink of extinction by the late 1960s, but subsequently underwent a natural recovery (Zimen and Boitani 1975; Gervasi et al. 2024). However, bottleneck legacies, such as low genetic diversity, high levels of inbreeding, and a significant genetic load, might still affect the long‐term survival of this population (Battilani et al. 2024). This vulnerable genomic status, along with the lack of proactive management actions, and the widespread presence of free‐ranging dogs, coupled with persistently high levels of poaching, heightens the risk of WDH for Italian wolves. Indeed, intensive non‐invasive genetic surveys have revealed alarmingly high WDH proportions at the local scale (Salvatori et al. 2019; Santostasi et al. 2021), raising further concerns on the potential impact of dog introgression. This is particularly relevant given the recent expansion of wolves into semi‐urban and urban environments where altered behaviors could affect wolf interactions with prey or toward humans, as well as relationships within packs (Zanni et al. 2023; Fardone et al. 2025; Di Bernardi et al. 2025). Therefore, understanding whether dog introgression might impact behavioral traits such as boldness, risk‐taking, or social tolerance is not only an evolutionary question but also a conservation and management priority.

The use of traditional genetic markers has proved effective in identifying recent hybrids across Europe (Caniglia et al. 2013; Harmoinen et al. 2021; Stronen et al. 2022). Then, the development of diagnostic SNP chips including thousands of markers allowed a greater resolution into the relevance of dog introgression (Galaverni et al. 2017; Pilot et al. 2018; Lobo et al. 2023). Indeed, Galaverni et al. (2017) found that admixed Italian wolves carried several genomic regions with significant excess of dog ancestry, likely resulting from introgression events that occurred up to 20 generations ago. However, because this study relied on a SNP chip covering only a subset of the genome, and primarily designed from known dog variation, its resolution may have been limited for accurately dating introgression events or detecting more complex evolutionary processes, such as selection on specific genomic regions. Whole‐genome sequencing data offers more resolution needed to investigate the temporal and functional dynamics of dog introgression at fine scale. This is extremely significant in the context of WDH, as while recent introgression—likely occurring in the past few generations—holds current management relevance, understanding historical introgression is critical to clarify its role in influencing the evolutionary trajectories of local wolf populations (Sarabia et al. 2025; Lobo et al. 2025).

The availability of well‐annotated dog reference genomes enables even deeper insight into the consequences of WDH on particular phenotypic traits (Lindblad‐Toh et al. 2005; Wang et al. 2021; Hörtenhuber et al. 2024). These resources have already been used to link specific mutations to morphological, physiological, and disease‐related traits in dogs, some of which could be subjected to introgression into wolves (Candille et al. 2007; Plassais et al. 2019; Dutrow et al. 2022). For example, there are several morphological traits found in wolves potentially indicative of WDH, such as melanism and dew claw (Ciucci et al. 2003; Caniglia et al. 2013; Galaverni et al. 2017), but these phenotypes do not necessarily reflect recent introgressions, as shown for melanism in North American wolves that traces back to ancient dog introgression events (Schweizer et al. 2018). Moreover, annotated dog genomes and the increasing research on canine behavioral genetics can offer a framework to explore the potential consequences of dog introgression on wild wolf behaviors (Kis et al. 2014; vonHoldt et al. 2017; MacLean et al. 2019; Shan et al. 2021; Tonoike et al. 2022; Morrill et al. 2022; Dutrow et al. 2022). Several genes have been linked to specific behavioral traits in dogs, including sociability (vonHoldt et al. 2017) and herding‐related behaviors (Dutrow et al. 2022; Jeong et al. 2025). Notably, some dog‐specific variants associated with behavior have also been identified as introgressed and under selection in the Iberian wolf population (Sarabia et al. 2025; Lobo et al. 2025). Although not all annotated genes are directly tied to behavior, functional annotations related to metabolic and physiological processes (Peel et al. 2022; Park et al. 2023; Ma et al. 2025) can offer valuable insight into the potential ecological and behavioral consequences of dog introgression in wild wolf populations.

In this study, we analyzed a total of 17 Italian wolf whole genomes, 14 of which were previously published (Fan et al. 2016; Battilani et al. 2024) and three, displaying atypical morphological traits potentially indicative of WDH, were newly sequenced. We merged these genomes with more than 100 worldwide‐distributed wolf and nearly 300 dog genomes, establishing a canid dataset well representative of both wild and domestic ancestries. Using multiple genome‐wide and local ancestry reconstruction approaches, we focused on the Italian wolf population to: (i) quantify the extent of dog ancestry, (ii) explore whether dog ancestry in the admixed wolves was higher than expected in behavior‐related genes, (iii) identify possible historical dog introgressions and verify whether these might be under putative selection.

## 2 Materials and Methods

### 2.1 Canid Genome Dataset

Tissue samples were collected from three peninsular Italian wolves (*Canis lupus italicus*) that were found dead in the Northern (2005–2008) and Central (2012) Apennines. All of them exhibited atypical morphological traits putatively indicative of WDH: two were melanic (i.e., W893, W1023), thus carrying the dog‐derived *K‐locus* (Candille et al. 2007; Anderson et al. 2009), which we confirmed using the analytical protocol developed by Caniglia et al. (2013); the third one (W1456) displayed the dew claw (i.e., a fifth, disarticulated digit in the hind legs), another dog‐derived trait associated with WDH (Ciucci et al. 2003; Galaverni et al. 2017). Sample storage, DNA extraction, library preparation and whole‐genome sequencing protocols followed Battilani et al. (2024). The 14 previously published Italian wolf genomes belonged to wolves sampled in the Central (*n* = 13) and Southern (*n* = 1) Apennines (Fan et al. 2016; Battilani et al. 2024). Four of these had been previously reported to be admixed with dogs based on individual genome‐wide ancestry proportion analyzes (Battilani et al. 2024). Moreover, we used genomic data from the ENA and NCBI GenBank public databases, to include wolves from across their entire species' range, together with a broad set of dog genomes belonging to medium‐large breeds that could potentially interbreed with wild wolves. We thus ensured a wide representation of dog and wolf ancestries, which was essential for identifying reliable reference individuals through genome‐wide analyzes and for subsequent local ancestry inference analyzes. Our final dataset consisted of 130 gray wolves ( *Canis lupus* ) (17 from Italy, 35 from other European countries, 30 from Asia, 48 from North America), 282 modern domestic dogs ( *Canis lupus familiaris* ) (representing 98 different breeds), and one coyote ( *Canis latrans* ) used as an outgroup (Table S1).

### 2.2 Quality Control, Assignment and Genotype Processing

We applied a quality control procedure on the sequencing reads, using FastQC (Andrews 2010) to check for possible issues such as low quality scores and anomalous GC content, and we used multiQC (Ewels et al. 2016) to visualize them. The reads were mapped onto the dog reference genome (CanFam3.1; Lindblad‐Toh et al. 2005) using the automated PALEOMIX BAM pipeline (Schubert et al. 2014), using the BWA ‘mem’ algorithm that is recommended for modern samples (Li and Durbin 2009), and setting the minimum mapping quality to 0 to retain all the reads in this step. Following this, we used SAMtools (Danecek et al. 2021) to remove non‐primary alignment reads (samtools view – F 256). We used GATK v 4.3.0.0 and referred to GATK Best Practice Workflow to call high quality genotypes (Van Der Auwera et al. 2013). Following Battilani et al. (2024), we applied the same two additional GATK tools for ‘hard filtering’ our genotypes and, subsequently, we used VCFtools (Danecek et al. 2011) for ‘soft filtering’ our genotypes. We applied those GATK and VCFtools filters on different datasets according to the assumptions of the downstream analyzes, with filters for each analysis provided in Table S2. Moreover, we used NgsRelate2 to identify and eventually remove closely related individuals (Hanghøj et al. 2019) applying thresholds of KING‐robust kinship ≥ 0.20, R0 ≤ 0.1, and R1 ≥ 0.5 (Waples et al. 2019). All the downstream analyzes were conducted on autosomes.

### 2.3 Genome‐Wide Ancestry Inference Analyzes

We explored patterns of genetic differentiation among samples using Principal Components Analysis (PCA) implemented in PLINK v 1.90b6.21 (Chang et al. 2015). To estimate individual admixture proportions, we used a maximum likelihood approach in ADMIXTURE v 1.3.0 (Alexander et al. 2009), which tests a given number of ancestries (*K*) for each individual. We conducted three sequential ADMIXTURE analyzes. In the first run, we used the full dataset and tested K values from 2 to 10 to distinguish admixed wolves (i.e., individuals assigned any proportion of dog ancestry at the best *K*), non‐admixed wolves (i.e., no assigned dog ancestry), and non‐admixed dogs (i.e., no assigned wolf ancestry). In the second run, we included all Italian wolves and one non‐admixed representative per dog breed, testing *K* = 2. This allowed us to validate the ancestry status of Italian wolves and to identify a reduced and balanced panel of non‐admixed dogs to serve as references. In the third run, we included all non‐admixed European wolves and the same set of dogs used in the second run, again testing *K* = 2. This analysis was used both to validate non‐admixed European wolves and to further confirm the status of the dog references. For the admixed Italian wolves detected in our ADMIXTURE analyzes, we estimated what generation ago the admixture occurred, ranging from F1 to backcross 3 (BC3w), using the GUI version of apoh (Garcia‐Erill et al. 2023).

To evaluate genome‐wide introgression between wolves and dogs, we tested for excessive derived allele sharing using D‐statistics, as implemented in ‘qpDstat’ from ADMIXTOOLS2 v 5.1 (Durand et al. 2011; Maier et al. 2023). We tested two different topologies in the form of (((P1, P2), P3), P4): (i) gene flow between each Italian wolf (X) and non‐admixed dog relative to non‐admixed Italian wolves (NWIT), using *Canis latrans* as the outgroup (OUT) (X, NWIT, DOG, OUT) to test for dog introgression within each Italian wolf relative to other non‐admixed Italian wolves; (ii) gene flow between each non‐admixed European wolf (X) and non‐admixed dog relative to all other non‐admixed European wolves (WEU) (X, WEU, DOG, OUT) to test for dog introgression within non‐admixed European wolves. The focal individual (X) was always excluded from P2 to avoid circularity in the analyzes. We chose to use the coyote as an outgroup because there is no ongoing gene flow or past introgression detected between wolves in western Eurasia and the coyote (Bergström et al. 2022). A negative D‐statistic score indicates gene flow between P2 and P3, and/or P1 and P4, while a positive D‐statistic score indicates gene flow between P1 and P3, and/or P2 and P4. Standard errors and z‐scores were obtained by block‐jackknife, following Durand et al. (2011) and Patterson et al. (2012), and a *z*‐score > 3 was considered evidence of significant gene flow (Durand et al. 2011).

### 2.4 Local Ancestry Inference Analyzes

To identify dog ancestry regions across the Italian wolf genomes, we applied admixfrog (Peter 2020) using a 10 kb bin size. Source populations representing non‐admixed dog and wolf populations were defined based on genome‐wide ancestry inference analyzes. We tested two scenarios. First, to assess recent introgression (i.e., hybridization events occurring in the past 6–7 generations, here represented by admixed Italian wolves obtained through paragraph 2.3), we tested each Italian wolf individual using all other non‐admixed Italian wolves and non‐admixed dogs as source populations. Second, to evaluate historical introgression (i.e., older hybridization events occurring before ‘recent introgression’) we used each Italian wolf (i.e., admixed and non‐admixed) and non‐admixed European wolf individuals using all other non‐admixed Italian wolves, non‐admixed European wolves, and non‐admixed dogs as source populations. We retained bin assignments with posterior probability > 0.9 for homozygous (here WOLF and DOG) and heterozygous (here DOGWOLF) states, reconstructing local ancestry profiles for each individual. To reduce false positives and focus on candidate historical introgression that is likely to have been maintained in the Italian wolf population, we kept only the top 10 percentile of dog regions (DOG and DOGWOLF) shared among Italian wolves in the second scenario, while absent in non‐admixed European wolves, and therefore potentially exclusive to the peninsular population. This conservative statistical threshold was chosen to validate introgressions consistent across the population, while excluding rare or individual‐specific signals that could result from stochastic recombination or recent admixture.

For our candidate historical dog introgressions, we performed additional analyzes using local D‐statistics and F_st_ to better validate the dog ancestry. First, we phased haplotypes using SHAPEIT2 (Delaneau et al. 2008), leveraging the dog recombination map from (Auton et al. 2013) and setting a window size of 0.5 Mb. We then used Dsuite (Malinsky et al. 2021) to estimate local D‐statistics through a window‐scan approach (window size = 100 kb; step size = 10 kb), retaining regions in the top 0.1 percentile for both df and *f*_*dM*_ statistics, using non‐admixed European wolves and dogs as source populations. Next, we estimated F_st_ through VCFtools (Danecek et al. 2011) using a window‐scan approach (window size = 100 kb; step size = 10 kb). We validated regions where all the Italian wolves showed substantially lower F_st_ from dogs than non‐admixed European wolves, retaining only those falling within the lowest 1 percentile of chromosome‐wide differences. We did not apply the shared‐regions filtering, the local introgression statistics, and F_st_ analyzes to the candidate recent introgressions from the first scenario, due to the limited statistical power provided by the reduced number of admixed individuals.

### 2.5 Behavior‐Related Genes Assessment and Shuffling

We developed an exploratory and targeted approach to evaluate if admixed Italian wolves exhibited under‐ or overrepresentation of dog ancestry in behavior‐related genes. To define candidate genes, we conducted a literature search of studies reporting genetic associations with behavioral traits in canids (Table S3). Because these associations are based largely on domestic dog studies, this gene set should be considered a tentative reference rather than a definitive catalog of wolf behavioral loci. We then used VCFtools (Danecek et al. 2011) to estimate per‐SNP F_st_ in order to detect the behavior‐related genes that contained highly differentiated SNPs (i.e., at least one SNP with F_st_ > 0.95) between non‐admixed Italian wolves and non‐admixed dogs. To generate a null distribution, we utilized the ‘shuffle’ feature (*n* = 1000 repetitions) from bedtools (Quinlan and Hall 2010). This method involved randomly shuffling genome‐wide regions matching the size (in bp) of the behavior‐related genes containing highly differentiated SNPs. We applied the ‘intersect’ feature from bedtools (Quinlan and Hall 2010) to intersect each shuffle and the highly differentiated behavior‐related genes with the local ancestry inference result from the first tested scenario of the ‘local ancestry inference analyzes’. In this context, we considered all the regions assigned to DOG and DOGWOLF, as our aim was to account for the total proportion of dog ancestry within admixed Italian wolves. Finally, we calculated the proportion of dog ancestry in both shuffles and highly differentiated genes, clustering them into 100 intervals of increasing dog ancestry to plot a normal distribution for admixed Italian wolves, with the number of shuffles per interval on the ‘y’ axis.

### 2.6 Selection Signatures Within Italian Wolves

We assessed signatures of selection at historical dog introgressions identified within the Italian wolves (i.e., admixed and non‐admixed). First, we calculated Tajima's D (Tajima 1989) in non‐overlapping windows of 50 kb using the software VCFtools (Danecek et al. 2011), and dog introgressions were identified as statistical outliers within the top 1 percentile of chromosome‐wide values. In the context of selection, positive Tajima's D values suggest balancing selection, whereas negative values may indicate positive selection. Next, we visualized haplotypes around outlier Tajima's D regions, expecting heterozygosity under balancing selection or homozygosity under positive selection. To further explore signs of balancing selection, we estimated Non‐Central Deviation (NCD) statistics which detect deviations in allele frequencies from neutrality and allow identifying genomic regions where multiple alleles may be maintained (Bitarello et al. 2018). Particularly, we computed NCD2 statistics to target long‐term balancing selection on historical dog introgressions using the ‘baselsr' package (Bitarello et al. 2018) in 50 kb windows and detecting the top 1 percentile outliers along the chromosome. To further explore signs of positive selection, we estimated the integrated haplotype score (iHS), a metric of extended haplotype homozygosity (EHH) that compares the ancestral and derived alleles at a given SNP. We detected outliers based on z‐score (α = 0.01) and the top 1 percentile of log‐transformed *p*‐values along each chromosome. For each of our historical dog introgressions with outliers identified in previous selection tests, we selected the top SNP and performed the single‐locus extended haplotype homozygosity (EHH) test, assessing the relationship between the frequency of a focal allele and the extent of surrounding linkage disequilibrium. Both iHS and EHH tests were used from the ‘rehh’ package (Gautier and Vitalis 2012).

### 2.7 TMRCA of Historical Dog Introgressions Under Positive Selection

We estimated the timing of historical dog introgressions using a Markov chain Monte Carlo (MCMC) approach implemented in STARTMRCA (Smith et al. 2018). This method relies on the decay of linkage disequilibrium between the selected allele and nearby sites to calculate the time to the most recent common ancestor (TMRCA), under the assumption of positive selection. Therefore, we focused on 1 Mb surrounding the SNPs with the highest iHS values and single‐locus EHH profiles within the historical dog introgressions under positive selection. For the reference panel, we used haplotypes from non‐admixed European wolves. We extracted the recombination rate for each historical dog introgression from the (Auton et al. 2013) recombination map and we tested two commonly used mutation rates for dogs and wolves: 4 × 10^−9^ (Skoglund et al. 2015) and 4.5 × 10^−9^ (Koch et al. 2019). We ran 10 independent MCMC chains, each comprising 50,000 iterations with a standard deviation of 10 for the proposal distribution. Posterior TMRCA distributions were generated from the final 10,000 iterations of each chain. TMRCA estimates were converted to years using a generation time of 4.4 years, representing the average of values derived from two independent studies (Vonholdt et al. 2008; Mech et al. 2016).

### 2.8 Gene Ontology

We performed gene ontology (GO) analyzes using STRING (Szklarczyk et al. 2023) *Canis lupus familiaris* database to investigate both functional enrichment and gene network clusters regarding all the historical dog introgressions validated through ‘local ancestry inference analyzes’. Genes within and downstream of these historical dog introgressions were annotated with GO terms related to biological processes, molecular functions, cellular components and local network clusters. We identified significantly enriched GO categories using false discovery rate (FDR) correction to ensure statistical robustness. To explore gene interactions, we set the default network size to a maximum of 10 interactors per gene and applied a required high confidence score of 0.7 to ensure the reliability of the predicted interactions. If we could not find a significant gene network, we reduced the required confidence score to the default value (medium confidence = 0.4).

## 3 Results

The three peninsular Italian wolves with putative WDH morphological traits had an average 25× coverage. Overall, the whole genomes we included in our dataset ranged between 4.8 and 52.2× coverage (Table S1). After aligning our total dataset of 413 genomes to the CanFam3.1 reference genome and the genotype‐calling procedure, we obtained 7,397,906 SNPs. We did not find any highly related pairs within the 17 Italian wolf individuals. However, to avoid any admixture and introgression inference biases, we excluded one individual that displayed two out of three relatedness indexes beyond the thresholds and had a low genotype quality. Additionally, we removed 2 European and 4 North American wolves due to their high relatedness.

### 3.1 Genome‐Wide Analyzes of Dog Ancestry in Italian Wolves

**FIGURE 1:**
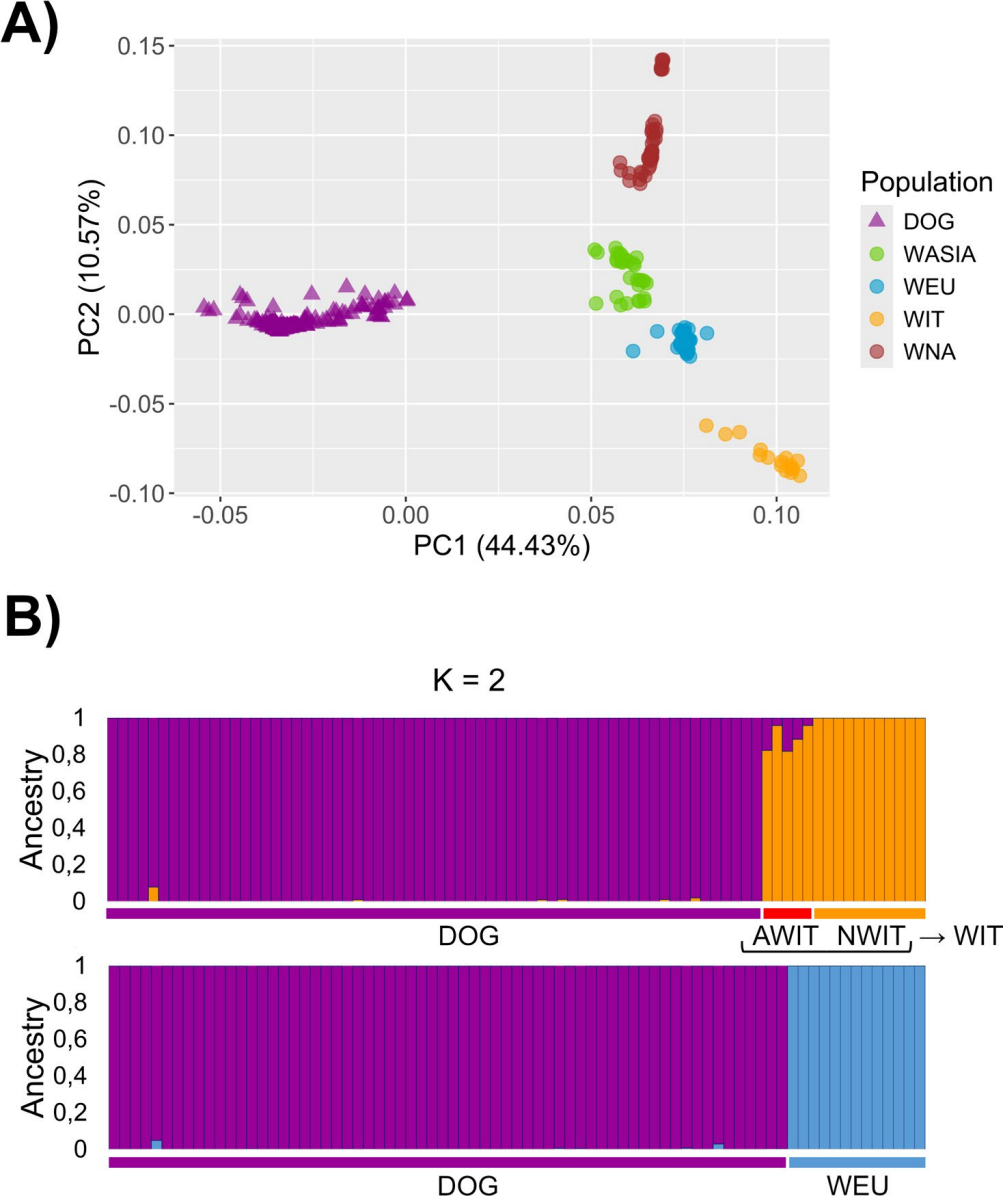
(A) Principal Component Analysis (PCA) showing patterns of genetic differentiation among samples, including mid‐large size dog breeds (DOG), Italian wolves (WIT), European wolves (WEU), Asian wolves (WASIA), and North American wolves (WNA). (B) ADMIXTURE analysis with K = 2 on a subset of samples to validate (i) admixed Italian wolves (AWIT) and non‐admixed Italian wolves (NWIT) (upper figure), (ii) non‐admixed WEU (lower figure), and obtain a reference panel of non‐admixed DOG crossing these two ADMIXTURE runs.

In the PCA analyzes, the first principal component explained most of the genetic variability (PC1: 44.43%) and separated wolves from dogs, whereas the second component (PC2: 10.57%) distinguished wolf populations at a continental scale, with Italian wolves that resulted the most differentiated from all the European wolves (Figure 1A). No individuals were positioned between dogs and wolves, suggesting that first‐generation hybrids (F1) were absent from the dataset. In the first ADMIXTURE analyzes, *K* = 10 was identified as the optimal K and, importantly, it was sufficient to discriminate between wolf and dog ancestry in each individual (Figure S1A). We identified 5 admixed Italian wolves, 11 non‐admixed Italian wolves and 13 non‐admixed European wolves (from Scandinavia) (Figure S1B). All the admixed Italian wolves showed more than 1% of dog ancestry (up to 20%), while 30 European wolves from other countries showed below 1%. Among these, only 13 Scandinavian wolves showed no dog ancestry.

The second and third rounds of ADMIXTURE confirmed the presence of dog ancestry in previously identified admixed Italian wolves, the absence of dog ancestry in previously identified non‐admixed European wolves, and a set of 53 reference dogs without any wolf ancestry (Figure 1B). Four out of five admixed Italian wolves corresponded to the individuals already identified by Battilani et al. (2024). Among the three Italian wolves with atypical morphological traits, only the individual with the dew claws resulted admixed (17.6% assigned dog ancestry at *K* = 4), while both melanic wolves were non‐admixed. The ‘apoh’ analyzes inferred that 4 out of 5 admixed Italian wolves were admixed 3–4 generations ago (i.e., one BC2w, one BC3w, and two intermediate between BC2w and BC3w) (Figure S2). The fifth admixed individual exhibited a pedigree distance (*d* = 0.13) that was closer to the independent pedigree distance (*d* = 0.07), suggesting that admixture might have occurred earlier than four generations ago. This roughly aligned with ADMIXTURE results based on the observed proportion of dog ancestry indicating an interbreeding event roughly occurred 6–7 generations ago (Table S4).

**FIGURE 2:**
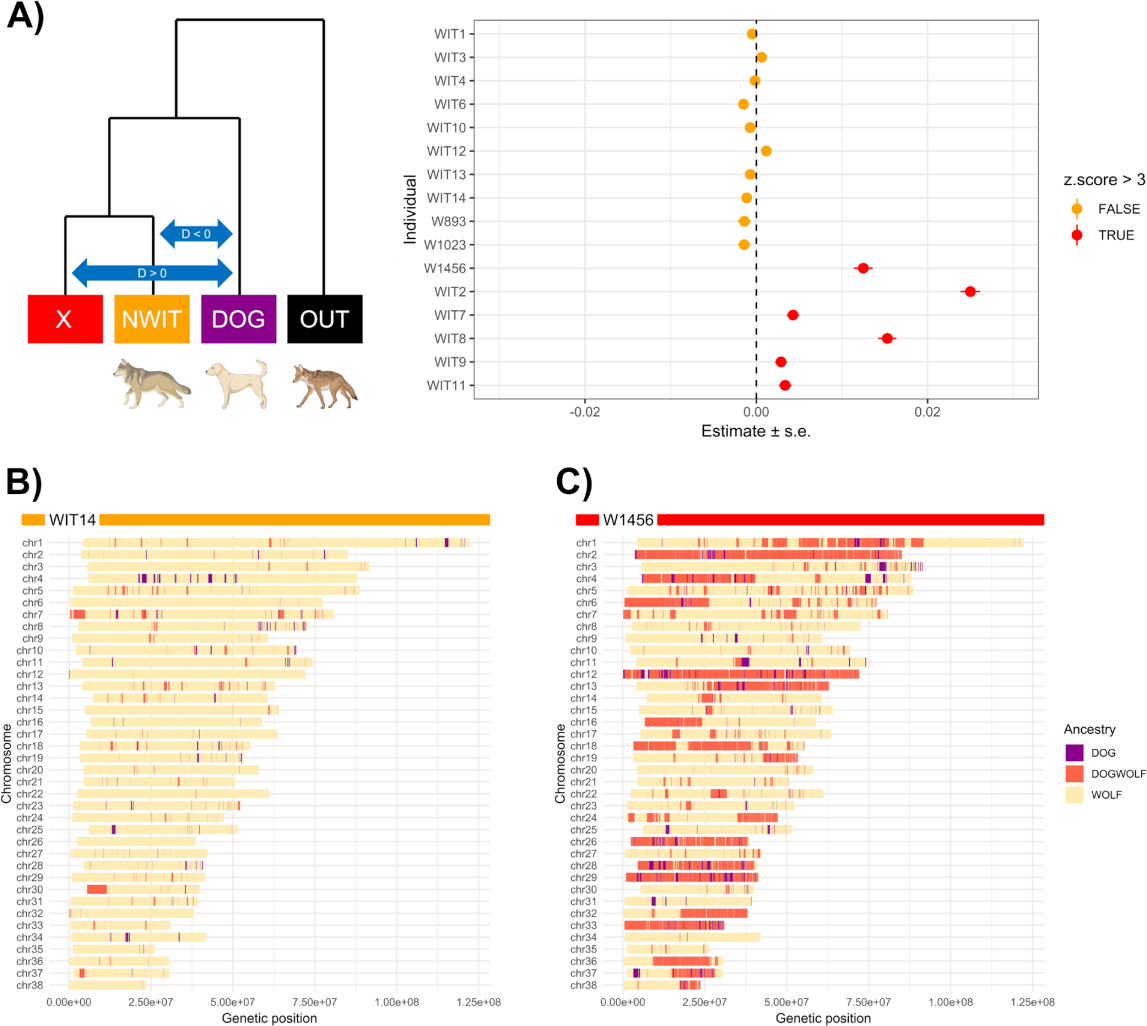
(A) Topology and results of the D‐statistics analysis testing for potential gene flow between each Italian wolf individual (X) and non‐admixed dogs (DOG) relative to non‐admixed Italian wolves (NWIT), using *Canis latrans* as an outgroup (OUT) ((X, NWIT), DOG, OUT). Individuals with a z‐score > 3 (red points) are considered to show excess allele sharing with DOG compared to NWIT. (B) Local ancestry inference analysis displaying the whole genomes of one non‐admixed Italian wolf (WIT14) and (C) one recently admixed Italian wolf (W1456).

Consistent with the PCA and ADMIXTURE analyzes, the five admixed Italian wolves showed significant derived allele sharing with dogs compared to non‐admixed Italian wolves (Figure 2A). We also identified an additional Italian wolf (i.e., WIT7) showing significant signs of derived allele sharing with dogs, which may indicate an older admixture event. Also consistent with the PCA and ADMIXTURE analyzes, the 2 melanic individuals were confirmed as non‐admixed. When testing for evidence of dog introgression in wolves across Europe, none of the 13 non‐admixed European wolves displayed significant allele sharing with dogs (Figure S3).

### 3.2 Local Ancestry Inference and Shared Dog Introgressions

**FIGURE 3:**
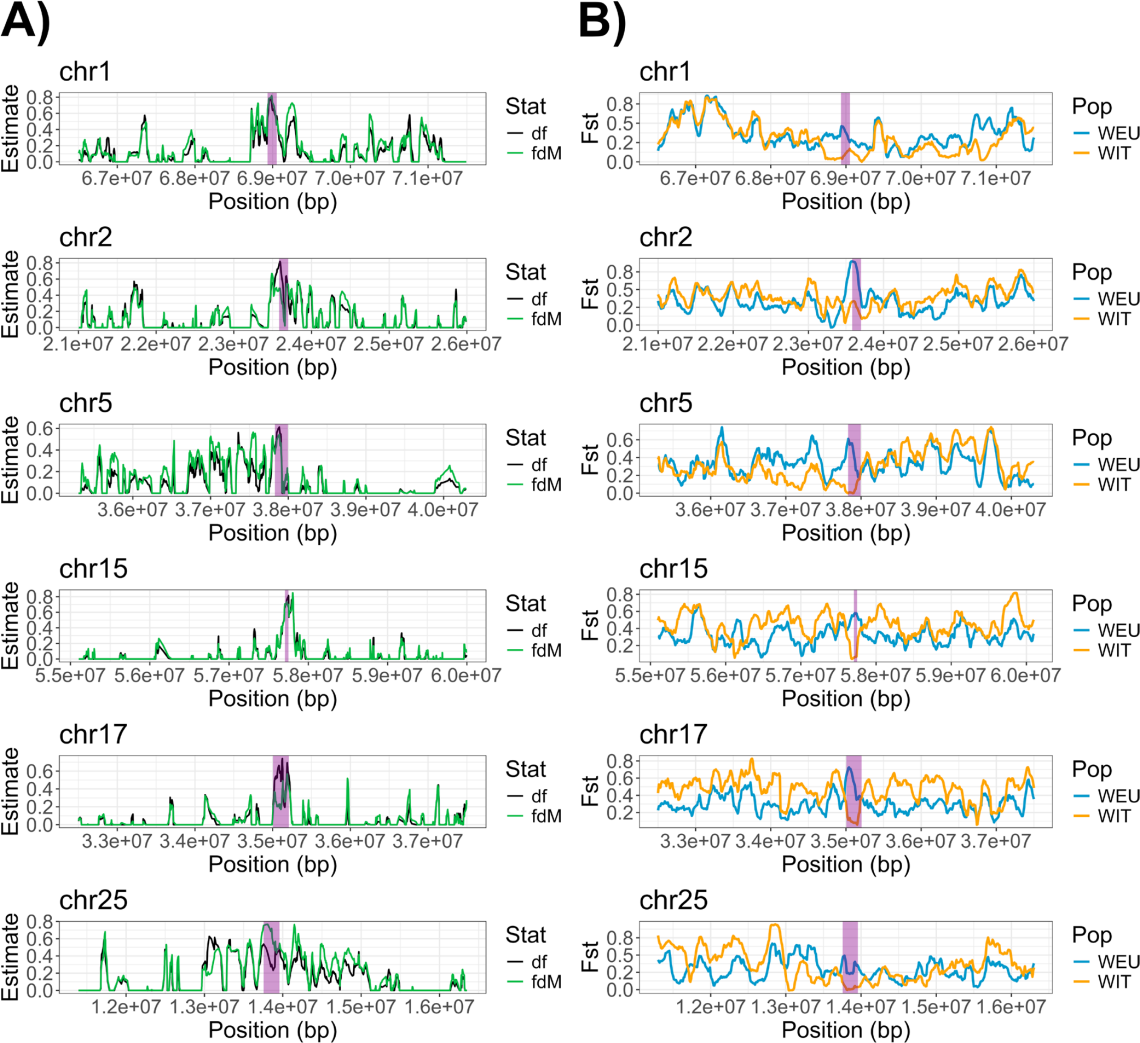
Validated introgressed regions in the top 10th percentile of Italian wolves (i.e., admixed and non‐admixed, WIT) shared regions, within a 5 Mb region surrounding each introgressed region. (A) df and *f*_*dM*_ local D‐statistics where a high df or *f*_*dM*_ value indicates evidence of gene flow between dogs and WIT. (B) *F*_*st*_ window‐scan results where a high *F*_*st*_ indicates high genetic differentiation compared to dogs. Purple‐shaded areas represent the top 0.1% outliers concordant across local D‐statistics (df and *f*_*dM*_) within each dog region and the top 1% outliers in the *F*_*st*_ window‐scan analysis.

Using local ancestry inference analyzes under our second scenario (i.e., assessing shared dog ancestry due to historical introgression in Italian wolves), we detected 22 candidate historical dog introgressions in Italian wolves. These regions covered a total of 2,453,884 bp and were absent in non‐admixed European wolves. Ten of the 22 regions overlapped with at least one gene, and four were found in the regulatory region of at least one gene; a total of 24 genes were therefore explored in greater detail. The local introgression validation using df and *f*_*dM*_ statistics confirmed that seven of such regions were indicative of dog introgression in Italian wolf genomes, exhibiting significant outliers. Six of these regions showed high differentiation between Italian wolves and non‐admixed European wolves, and low differentiation between Italian wolves and dogs, as demonstrated by top outliers in the F_st_ window‐scan analyzes (Figure 3). For these reasons, we proceeded to perform selection signature analyzes on them. Additionally, we confirmed that both melanic wolves had dog ancestry detected in the *K‐locus* region, despite being identified as non‐admixed with our genome‐wide approaches.

### 3.3 Dog Ancestry in Targeted Behavior‐Related Genes

**FIGURE 4:**
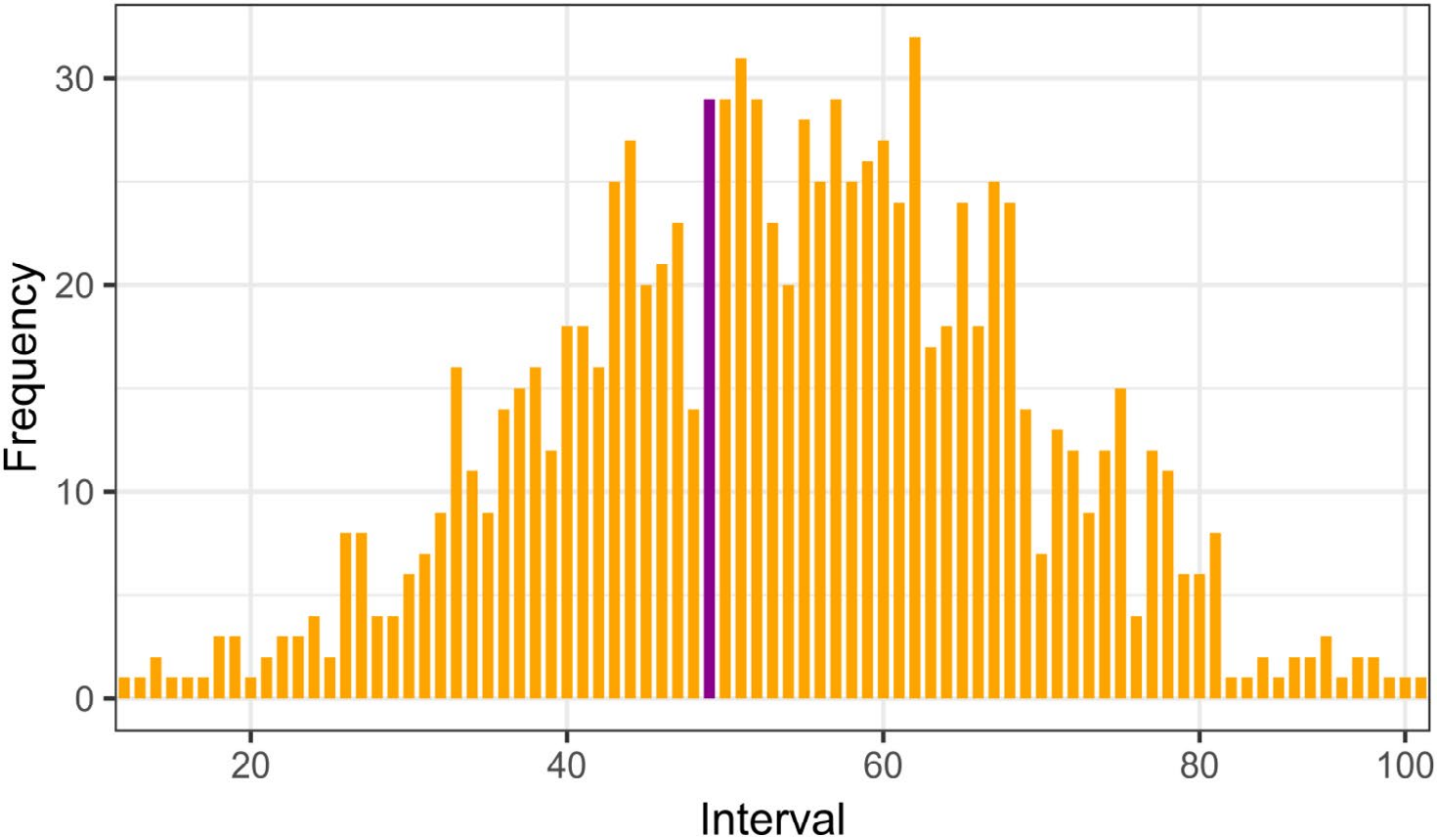
Normal distribution of 1000 genome shuffles for admixed Italian wolves, based on the size of highly differentiated behavior‐related genes and their dog ancestry. The x‐axis represents intervals of increasing dog ancestry, while the y‐axis shows.

From the literature, we identified 599 candidate genes previously associated with behavioral traits in canids (Table S3). Our per‐SNP F_st_ scan between non‐admixed Italian wolves and non‐admixed dogs found 6927 top SNPs (Fst > 0.95) within 340 of these 599 behavior‐related genes. The behavior‐related gene shuffling analyzes conducted on admixed Italian wolves showed that the proportion of dog ancestry in the highly differentiated behavior‐related genes did not differ from expectations under neutrality. This indicates we failed to find significant under‐ or overrepresentation of dog ancestry in behavior‐related genes compared to random genome shuffles (Figure 4).

### 3.4 Signs of Balancing and Positive Selection in Historical Dog Introgressions

**FIGURE 5:**
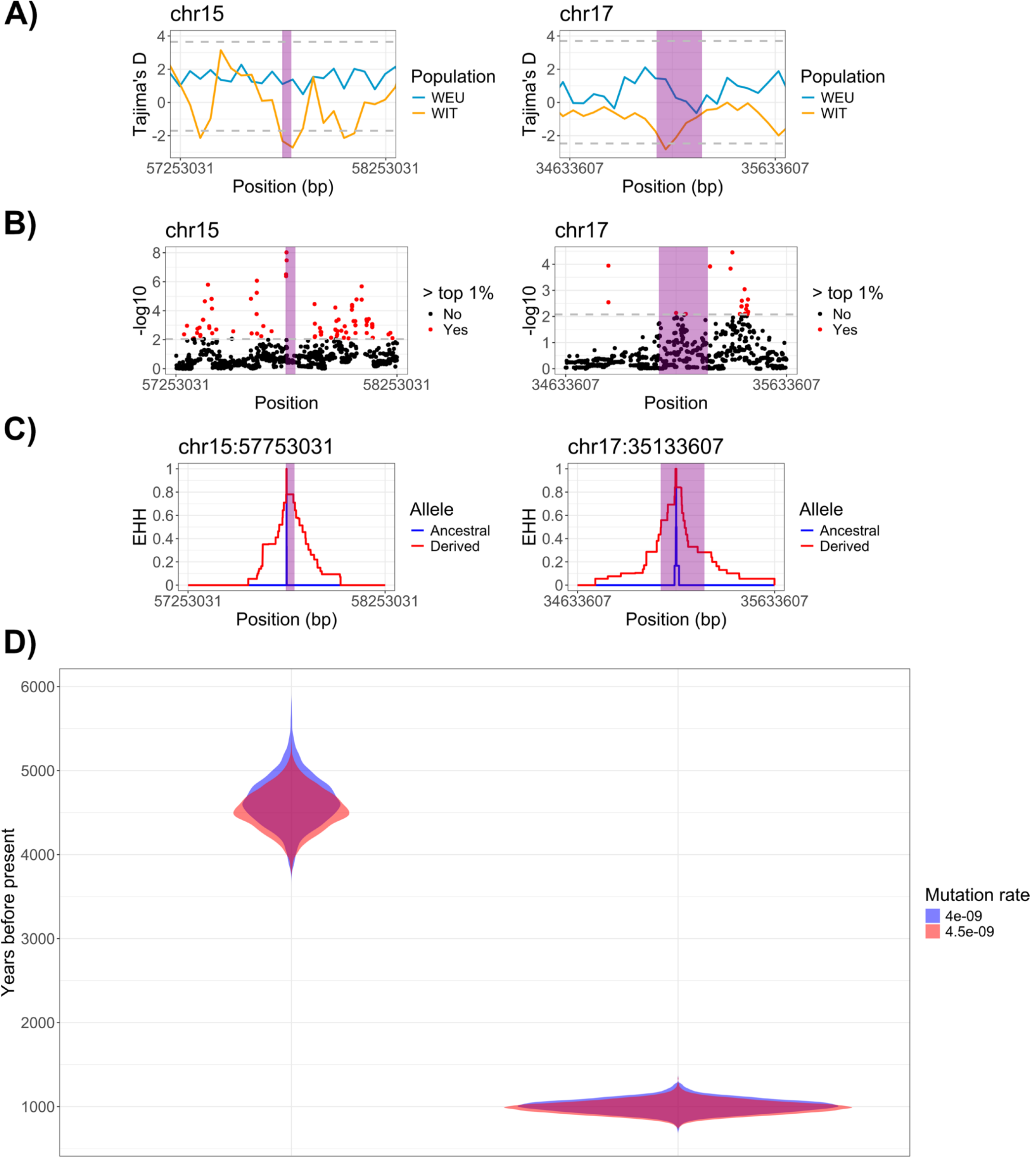
One Mb around the two introgressed dog regions within the Italian wolf population with positive selection signatures (purple shaded areas) showing: (A) Tajima's D estimates, based 50 kb windows. Dashed gray lines represent top 1 percentiles for positive and negative estimates; (B) top 1 percentile outliers of iHS estimates; (C) EHH around top iHS outliers; (D) TMRCA for the timing of selection centered on iHS top outlier based on two different mutation rates (Skoglund et al. 2015; Koch et al. 2019).

We identified historical dog introgressions exhibiting patterns consistent with selection signatures. We found one region (chr2:23583224–23699763) with significant positive Tajima's D values, suggesting possible balancing selection (Figure S4A), in all the Italian wolves. Additionally, three dog introgressions within Italian wolves (chr15:57654252–57793236; chr17:35055609–35277373; chr25:13578063–13957991) exhibited significant negative Tajima's D values, indicating possible positive selection in these tracts (Figures 5A and S5). Using NCD statistics, the dog introgression identified in all Italian wolves (chr2:23583224–23699763) contained a top outlier within the top 1 percentile for NCD2 (Figure S4C). When using iHS to evaluate the haplotype scores of SNPs within the dog introgressions for Italian wolves, only chr15:57654252–57793236 and chr17:35055609–35277373 contained SNPs that were significant outliers by both z‐score and were in the top 1 percentile (Figure 4B). Analyzes of single‐locus EHH on the top outliers in these regions revealed patterns consistent with positive selection (Figure 5C).

### 3.5 Timing of Historical Dog Introgressions

We estimated the introgression timing and spread into the population of two historical dog introgressions exhibiting multiple signatures of positive selection. Using two mutation rates, TMRCA for the region surrounding chr15:57753031 ranged from 1031 to 1054 (95% CIs: 942–1167) generations ago, while for the region surrounding chr17:35133607 it ranged from 224 to 230 (95% CIs: 196–260) generations ago. Averaging the two mutation rates, and assuming a mean generation time of 4.4 years for wolves (Vonholdt et al. 2008; Mech et al. 2016), TMRCA for the chr15: 57753031 region was 4587 (95% CIs: 4161–5032) years ago, and for the chr17:35133607 region 998 (95% CIs: 871–1133) years ago (Figure 5D).

### 3.6 Functional Enrichments and Gene Networks

Within the six historical dog introgressions identified for Italian wolves, eight genes were classified with high confidence and one with medium confidence (Table S5; Figure S6). The top outlier SNP for positive selection on chr15:57654252–57793236, found with iHS and confirmed by EHH, was 193 kb upstream of FSTL5, a gene involved in the regulation of heparan sulfate proteoglycans (HSPGs), cellular membrane composition and ion transport mechanisms. The top outlier SNP for positive selection on chr17:35055609–35277373, found with iHS and confirmed with EHH, was located within NPHP1, which is associated with photoreceptor cell development and ciliary functions. This introgressed region also included BUB1, TPC3 and ACOXL, genes enriched for mitotic progression and checkpoint control mechanisms, modulation of synaptic activity in GABAergic pathways, and fatty acid beta‐oxidation, respectively. The gene ATP8A2, located within chr25:13578063–13957991 was identified as positively selected only by Tajima's D and was enriched for ATP‐dependent lipid transport across membranes. The gene CAMK1D (chr2:23583224–23699763), identified under balancing selection by Tajima's D and exhibiting top 1 percentile NCD2 outlier, participates in calcium ion binding, tricellular tight junction and positive regulation of regulatory bursts. Among genes within dog introgressions without significant selection signatures, EPB41L2 (downstream of chr1:68933941–69051712) was the only one with medium‐confidence enrichment, contributing to cytoskeletal organization and synaptic signaling. Moreover, COX10 (chr5:37827522–37994324) was linked to heme metabolism and the assembly of respiratory chain complex IV.

## 4 Discussion

The introgression of dog ancestry represents a relevant conservation threat due to its potential to alter the behavior, ecology, and evolution of wolf populations (Leonard et al. 2013; Hindrikson et al. 2017; McFarlane and Pemberton 2019; Ottenburghs 2021). Cues of introgressive WDH might be derived from some atypical morphological traits occasionally detected in admixed wolves (Caniglia et al. 2013; Galaverni et al. 2017), even though these cannot always reflect admixture or recent WDH (Stronen et al. 2025). Quantifying the extent of dog introgression using whole genomes and assessing its potential impact on phenotypic traits other than morphology, however, remains largely unexplored. We tried to address this knowledge gap by analyzing whole‐genome data from a sample of 17 Italian wolves.

Genome‐wide ancestry analyzes confirmed that WDH is ongoing in Italy, as we identified five recently admixed individuals (3–7 generations before sampling) out of 17. We estimated these admixed individuals resulted from interbreeding events that occurred between 1980 and 1999, a timing concordant with the hypothesis of a hybridization peak during population recovery in peninsular Italy (Lucchini et al. 2002; Fabbri et al. 2007; Galaverni et al. 2017). We didn't detect recently admixed European wolves from other countries representing our dataset (i.e., Croatia, Greece, Norway, Portugal, Spain). Despite 17 European wolves exhibiting < 1% of dog ancestry, we selected the 13 Scandinavian wolves with no dog ancestry as our reference population. Their suitability as reference wolves was supported by both the broad representation of our dog panel and previous research demonstrating their minimal dog ancestry and distinct genetic background (Smeds et al. 2021). Although the Scandinavian wolf population is known to be strongly inbred, it derives from the Finnish‐Russian (i.e., Karelian) lineage and shows evidence of bidirectional gene flow with that population, making it phylogenetically relevant within the broader Eurasian wolf context (Ellegren et al. 1996; Linnell et al. 2005; Smeds et al. 2021). Nevertheless, we acknowledge that the population's reduced genetic diversity and extended linkage disequilibrium might limit the resolution of local ancestry inference and could lead to underestimation of rare or fragmented introgressed tracts.

Among the three Italian wolf individuals displaying atypical morphological traits, only the one displaying dew claws showed a clear genome‐wide signature of recent admixture (i.e., 3–4 generations ago). Differently, the two melanic wolves did not exhibit any apparent genomic admixture, though local ancestry inference revealed that the region containing the *K‐locus* was of dog origin. This supports the hypothesis that the melanic variant was introgressed in Italian wolves through historical WDH events, as also suggested by previous studies based on a 170 k SNP dataset (Galaverni et al. 2017). A similar pattern was reported in North American wolves, where the *K‐locus* melanic mutation was estimated to be introgressed from dogs ~1600–7200 years ago (Schweizer et al. 2018) and has since increased in frequency due to positive selection, likely through enhanced camouflage in dark woodlands and immunity to distemper virus (Schweizer et al. 2018; Cubaynes et al. 2022). Nonetheless, it cannot be excluded that the *K‐locus* variant was already segregating at low frequency in wolves prior to domestication, as suggested by its detection in ancient pre‐domestication wolves (Bergström et al. 2022), thus our results underscore the need for further investigations using larger genomic datasets of melanic wolves and dogs to clarify the origin and spread of melanism.

Local ancestry inferences revealed that recently admixed Italian wolves did not show significant over‐ or underrepresentation of dog ancestry in highly differentiated behavior‐related genes. These findings suggest that, in the admixed wolves we sequenced, such genes did not show particular permeability or resistance to recent dog introgression. While it cannot be excluded that our results might have been due to random recombination, we do not find evidence to indicate that dog ancestry at these loci does confer a significant advantage. However, these findings should be interpreted with caution. First, the number of admixed individuals in our dataset was limited. Second, the gene set was derived from domestic dog studies and might not directly translate to wild wolf behavior. Finally, no behavioral phenotypes were available for the wolves we analyzed, leaving the functional impact of these genes in natural settings untested.

In addition to recent introgressions, we detected six historical dog introgressions in Italian wolves, located in genes whose functional enrichment suggests potential effects on physiology and behavior. Two of these historical dog introgressions exhibited multiple signatures of positive selection, allowing us to estimate their timing using the approach described by Smith et al. (2018). One region, located 193 kb upstream of the FSTL5 gene, was estimated to have introgressed ~4500 years ago, during the Bronze Age—a period when dogs were already integrated into human society, as shown by their frequent presence in burial sites (Bartosiewicz 2013; Morey and Jeger 2022). Consistently, a recent genomic study has reported dog ancestry dated from the Bronze Age in the now extinct Sicilian wolf (Ciucani et al. 2023), further supporting the occurrence of WDH in Italy during that time. FSTL5 is associated with neuronal and membrane signaling in dogs (Lisboa et al. 2019) and has been linked to logical versus affective orientations in humans (Kao et al. 2023). Because these associations point to a possible neurobehavioral role, one tentative interpretation is that dog ancestry at FSTL5 might influence cognitive or emotional regulation, potentially promoting more reactive responses to human presence. The second introgressed region is located within the NPHP1 gene and was dated to ~1000 years ago, a period coinciding with the Late Middle Ages and the sociopolitical transformations following the barbarian invasions of the Italian Peninsula. In this period dogs were highly valued, as evidenced by the first known breed classification recorded in the *Leges Barbarorum* (Iuffrida 2012). Given that NPHP1 is crucial for photoreceptor structure and visual function (Datta et al. 2021), this introgressed fragment might derive from medieval working dogs that were artificially selected to perform tasks such as hunting, herding, or guarding, where enhanced visual acuity or motion detection could have been advantageous. In addition to NPHP1, this introgressed region also includes BUB1, TPC3, and ACOXL, genes involved in cell cycle regulation, synaptic signaling, and lipid metabolism, respectively. Although not under the strongest selection signal, their retention may reflect subtle neurophysiological advantages, such as enhanced neural modulation or energy metabolism.

For historical dog introgressions where we did not estimate exact timing, but which occurred more than seven generations ago (i.e., our arbitrary threshold for distinguishing recent from historical introgression), we detected one region exhibiting multiple balancing selection signatures, as indicated by a top outlier in Tajima's D and a top 1 percentile NCD2 window. This region includes CAMK1D, a gene encoding for a key modulator of tumor‐intrinsic immune resistance in humans (Volpin et al. 2020), which might be beneficial for wolves exposed to high levels of carcinogenic substances produced by anthropic activities (Kravchenko et al. 2015; Madia et al. 2019). We finally identified an additional historical dog introgression showing a positive selection sign, but solely based on Tajima's D. This introgressed region includes ATP8A2, a gene implicated in neural plasticity (Xu et al. 2012), and associated with ‘excitability’ in a GWAS leveraging behavioral and genomic data of more than 1000 dogs (MacLean et al. 2019). The limited size of our historical dog introgressions under multiple selection signatures (~100–200 kb) would seem to exclude the role of the last historical bottleneck, experienced by Italian wolves in the late 1960s, in explaining these patterns (Battilani et al. 2024). However, a single Tajima's D outlier might not exclusively reflect selection but could also result from population contractions that occurred over the past few centuries. Therefore, future studies should validate the selection signatures that we identified, for instance by implementing demographic simulations.

We also identified two historically introgressed regions that did not show clear signatures of selection but displayed pronounced permeability to dog ancestry. For instance, genes such as EPB41L2 and COX10, located within these regions, may play roles in responses to environmental stressors or in enhancing energy efficiency (Schüll et al. 2015; Voges et al. 2024). While these hypotheses remain untested, all historically introgressed regions detected in our study warrant functional investigation to assess their potential phenotypic relevance. Such analyzes should ideally be conducted using larger sample sizes and more comprehensive panels of admixed and non‐admixed wolves.

Recent studies on Iberian wolves using whole‐genome data have similarly reported dog introgressions dating back to ancient times (Sarabia et al. 2025; Lobo et al. 2025). These results are consistent with earlier work, reinforcing the hypothesis that WDH is not only a contemporary conservation issue but a long‐standing anthropogenic impact capable of shaping the genomic landscape of wolf populations and, consequently, influencing their evolutionary trajectories (Skoglund et al. 2015; Pilot et al. 2018; Bergström et al. 2022). Notably, while the specific genes revealing dog introgression differ between Iberian and Italian wolves, functional enrichments converge on neurodevelopmental, immune, and metabolic pathways, which might affect behavior and adaptability to human‐modified environments (Sarabia et al. 2025; Lobo et al. 2025). This suggests that the outcomes of WDH are likely influenced by local ecological conditions, selective pressures, and population‐specific genomic architectures, all of which may have been disrupted or altered by anthropogenic activities. Moreover, introgressed regions differed between the two studies focusing on Iberian wolves, suggesting that fundamental analytical approaches, such as local ancestry inferences, can strongly affect the detected outcomes as well. This emphasizes the need for local‐scale, methodologically consistent and tailored genomic investigations to fully understand the possible evolutionary and conservation implications of dog introgression in wolves.

## 5 Conclusion

This study provides the first whole‐genome investigation on dog introgression in the Italian wolves. Using multiple genome‐wide and local ancestry inference approaches, we detected varying levels of genome‐wide dog ancestry in admixed wolves. Additionally, we explored an innovative approach to assess recently introgressed dog ancestry on behavior‐related genes, indicating that such genes were not particularly permeable to recent dog introgression. We also identified historical dog introgressions in Italian wolves, with functional enrichments that could be interpreted in the context of physiological, ecological and behavioral effects. These historical introgressions most likely represent the legacy of long‐standing human‐caused gene flow between Italian wolves and dogs, and might have affected not only the Italian wolf genomic make‐up but also its interactions with natural and anthropogenic environments. We showed that multiple genomic approaches can represent an effective tool to uncover the complex nature of dog introgression, which is particularly relevant in genetically eroded and isolated populations. While recent introgression has direct management implications due to its potential spread within the population, historical introgression is not actionable management‐wise. Yet, it remains relevant to investigate, as it might have shaped the evolutionary trajectories and adaptive potential of the species to human‐modified environments. In this context, understanding the behavioral and fitness‐related implications of introgressed dog ancestry is essential for preserving the genetic integrity of Italian wolves and managing their coexistence with humans. Future studies incorporating larger datasets of wild and admixed individuals, and combining whole‐genome with transcriptomic and epigenomic analyzes, will help elucidate the possible adaptive role and the potential phenotypic effects of introgressed dog variants.

## Author Contributions

**D. Battilani:** conceptualization (lead), formal analysis (lead), visualization (lead), writing – original draft (lead), writing – review and editing (lead). **J. Ramos Madrigal:** conceptualization (equal), writing – review and editing (equal). **L. M. Hennelly:** conceptualization (equal), writing – review and editing (equal). **S. Gopalakrishnan:** conceptualization (equal), resources (equal), writing – review and editing (equal). **C. Vernesi:** writing – review and editing (equal). **F. Mattucci:** writing – review and editing (equal). **E. Fabbri:** conceptualization (equal), resources (equal), writing – review and editing (equal). **P. Ciucci:** conceptualization (equal), resources (equal), writing – review and editing (equal). **R. Caniglia:** conceptualization (equal), resources (equal), writing – review and editing (equal).

## Funding

This work was supported by Sapienza Università di Roma (Grants DOT1326JZS‐17 and CUP B85F21005360001), European Union‐NextGenerationEU National Biodiversity Future Center and Istituto Superiore per la Protezione e la Ricerca Ambientale (ISPRA).

## Conflicts of Interest

The authors declare no conflicts of interest.

## Supporting information


**Table S1:** Whole‐genome dataset. Yellow = related individual removed from the dataset; orange = non‐admixed Italian wolf; red = admixed Italian wolf; blue = non‐admixed European wolf; purple = non‐admixed dog; green = outgroup.

**Table S2:** Genotype filtering summary for each kind of analyzes.

**Table S3:** List of genes associated with behavioral traits in dogs and captive wolves, tested using the ‘Behavior‐related genes assessment and shuffling’ approach.

**Table S4:** ‘apoh’ output for each WIT individual. Admixture pedigree compatibility is measured as the ‘distance’ between the paired ancestries expected under the ‘indipendent’ pedigree and the estimated ones. If pedigree 1 or 2 exhibits a lower value compared to the ‘indipendent’ one than the individual is recently admixed (colored in red). We also highlighted one individual non‐recently admixed but with close distances (in yellow).

**Table S5:** Gene ontology network results using STRING on genes within introgressed and validated regions in admixed Italian wolves (i.e., gene PREX2), and all the Italian wolves (the other genes). Green = functional enrichment for the candidate gene.

**Figure S1:** (A) Cross‐validation errors estimated for each number of cluster (*K*) in the first ADMIXTURE run. (B) ADMIXTURE plots based on the whole dataset, with *K* = 2, *K* = 5, and *K* = 10, representing dogs (DOG), Italian wolves (WIT), European wolves (WEU), Asian wolves (WASIA), and North American wolves (WNA).

**Figure S2:** Most likely admixture pedigree (Pedigree 1) compared to the indipendent ancestries pedigree for each recently admixed individual as a result of ‘apoh’ analyzes.

**Figure S3:** Results of the f4‐statistic analysis testing for potential gene flow between (i) each non‐admixed European wolf individual and (ii) other non‐admixed European wolves (NWIT) or (iii) dogs without Italian and European wolf ancestry (DOG), with (iv) *Canis latrans* used as an outgroup (OUT). Individuals with a z‐score > 3 are considered significantly introgressed genome‐wide.

**Figure S4:** (A) Tajima's D estimate of 1 Mb region surrounding the validated introgressed region (purple‐shaded areas) that exhibit signs of balancing selection on the entire subset of Italian wolves. The dashed gray lines represent top 1 percentiles for positive and negative Tajima's D estimates. (B) NCD2 statistics estimates of 1 Mb region surrounding the same validated introgressed region (purple‐shaded areas) that exhibit signs of balancing selection. The dashed gray lines represent top 1 percentiles. (C) Painted haplotypes for the same introgressed validated region are shown (DOG = non‐admixed dogs; WIT = admixed & non‐admixed Italian wolves sharing the region; WEU = non‐admixed European wolves). ‘R’ and ‘A’ refer to reference and alternate alleles for each SNP.

**Figure S5:** Haplotype paintings for the three validated introgressed regions exhibiting signs of positive selection (DOG = non‐admixed dogs; WEU = non‐admixed European wolves; WIT = admixed and non‐admixed Italian wolves sharing the region). ‘R’ and ‘A’ refer to reference and alternate alleles for each SNP.

**Figure S6:** Significant gene networks obtained with STRING for the genes on the validated introgressed regions on the top 10th percentile of non‐admixed & admixed Italian wolves (WIT) shared regions. Each network confidence is specified. The purple circle surrounds the candidate gene.

**Supinfo S1.** ece372508‐sup‐0012‐SupinfoS1.docx.

## Data Availability

All the codes used in this study have been submitted to Zenodo (https://doi.org/10.5281/zenodo.17464103). Raw DNA sequence reads of whole genomes generated in this study have been submitted to the European Nucleotide Archive (ENA) (Accession: PRJEB102393; Secondary Accession: ERP183791).
