# Correction to “Whole‐Genome Data to Investigate Recent and Historical Dog Introgression Patterns in Italian Wolves”

**DOI:** 10.1002/ece3.72817

**Published:** 2025-12-17

Battilani, D., J. Ramos Madrigal, L. M. Hennelly, et al. 2025. “Whole‐Genome Data to Investigate Recent and Historical Dog Introgression Patterns in Italian Wolves.” *Ecology and Evolution* 15, no. 12: e72508. https://doi.org/10.1002/ece3.72508.

In the published article, the caption of Figure 4 was incomplete. The full caption should read as follows:

Figure 4. Normal distribution of 1000 genome shuffles for admixed Italian wolves, based on the size of highly‐differentiated behavior‐related genes and their dog ancestry. The *x*‐axis represents intervals of increasing dog ancestry, while the *y*‐axis shows the frequency of shuffled values within each interval. The purple bar marks the interval corresponding to the observed dog ancestry of the behavior‐related genes.”

We apologize for this error.

