# Supplementary figures and images for: Whole‐Genome Data to Investigate Recent and Historical Dog Introgression Patterns in Italian Wolves
Source: Ecol Evol. 2025 Nov 27;15(12):e72508. doi: 10.1002/ece3.72508 (PMC12658625; doi:10.1002/ece3.72508)

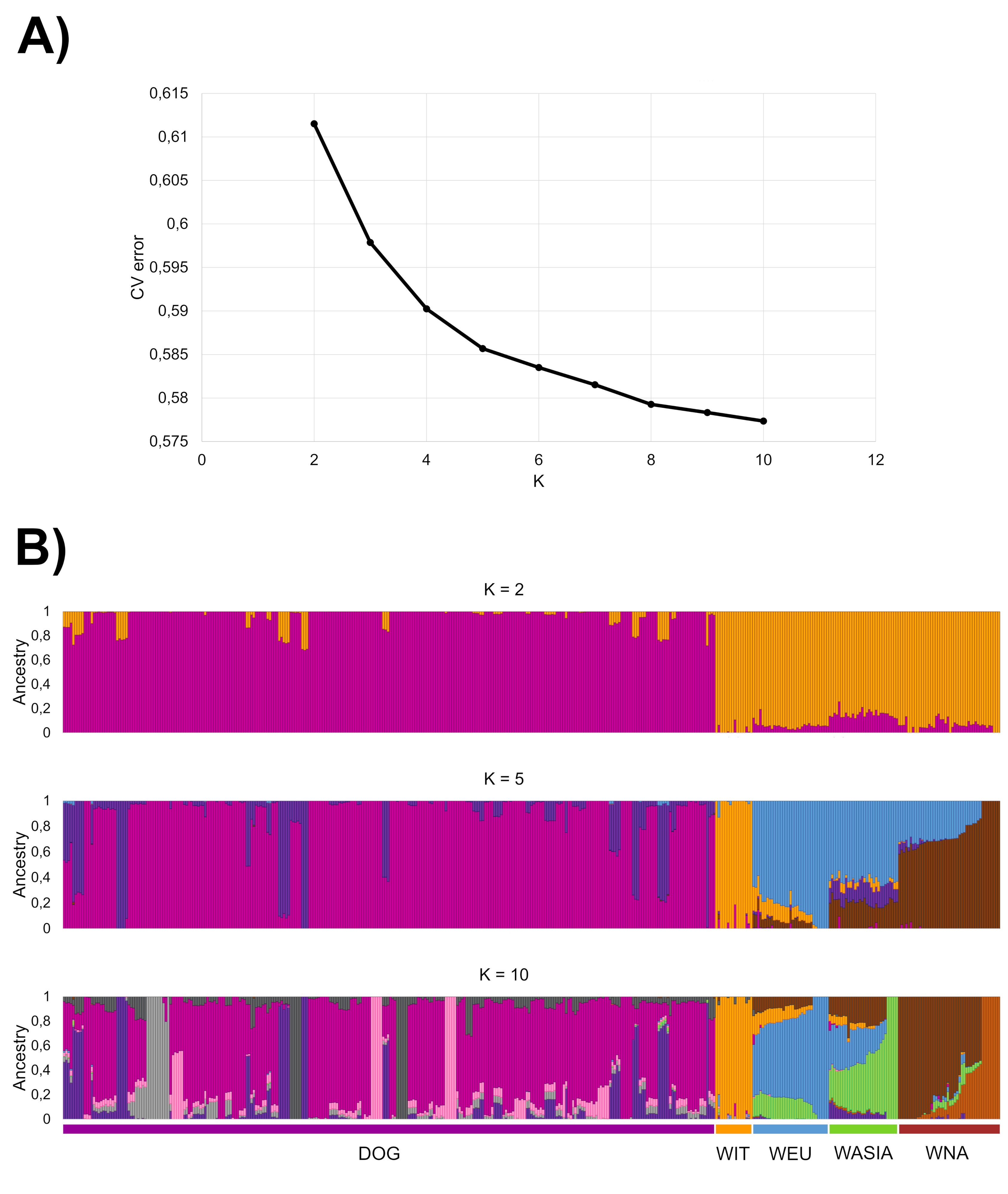

Supplement: Supplementary file 6 — Figure S1: (A) Cross‐validation errors estimated for each number of cluster (K) in the first ADMIXTURE run. (B) ADMIXTURE plots based on the whole dataset, with K = 2, K = 5, and K = 10, representing dogs (DOG), Italian wolves (WIT), European wolves (WEU), Asian wolves (WASIA), and North American wolves (WNA). [file ECE3-15-e72508-s002.jpeg]

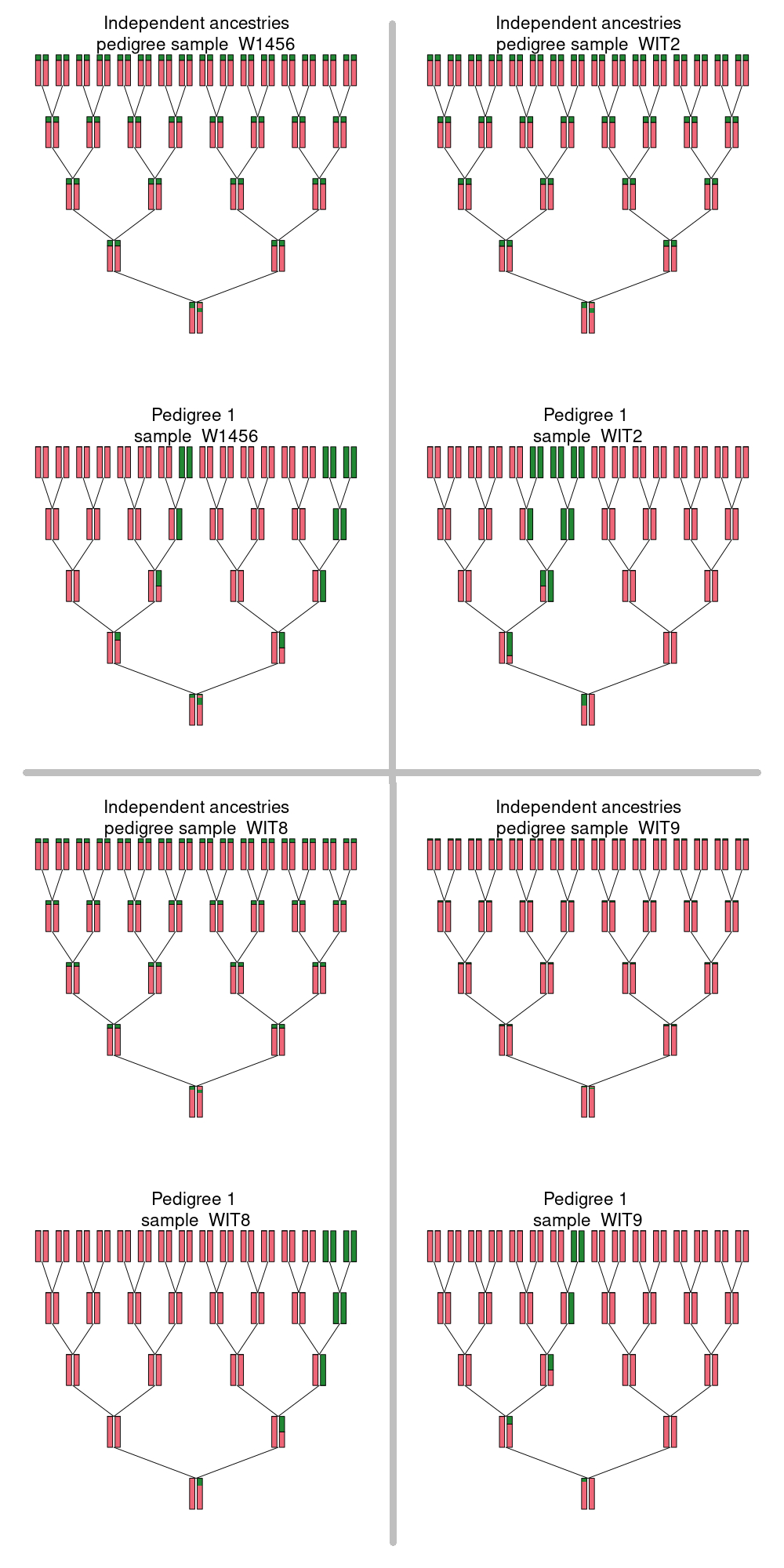

Supplement: Supplementary file 7 — Figure S2: Most likely admixture pedigree (Pedigree 1) compared to the indipendent ancestries pedigree for each recently admixed individual as a result of ‘apoh’ analyzes. [file ECE3-15-e72508-s005.jpeg]

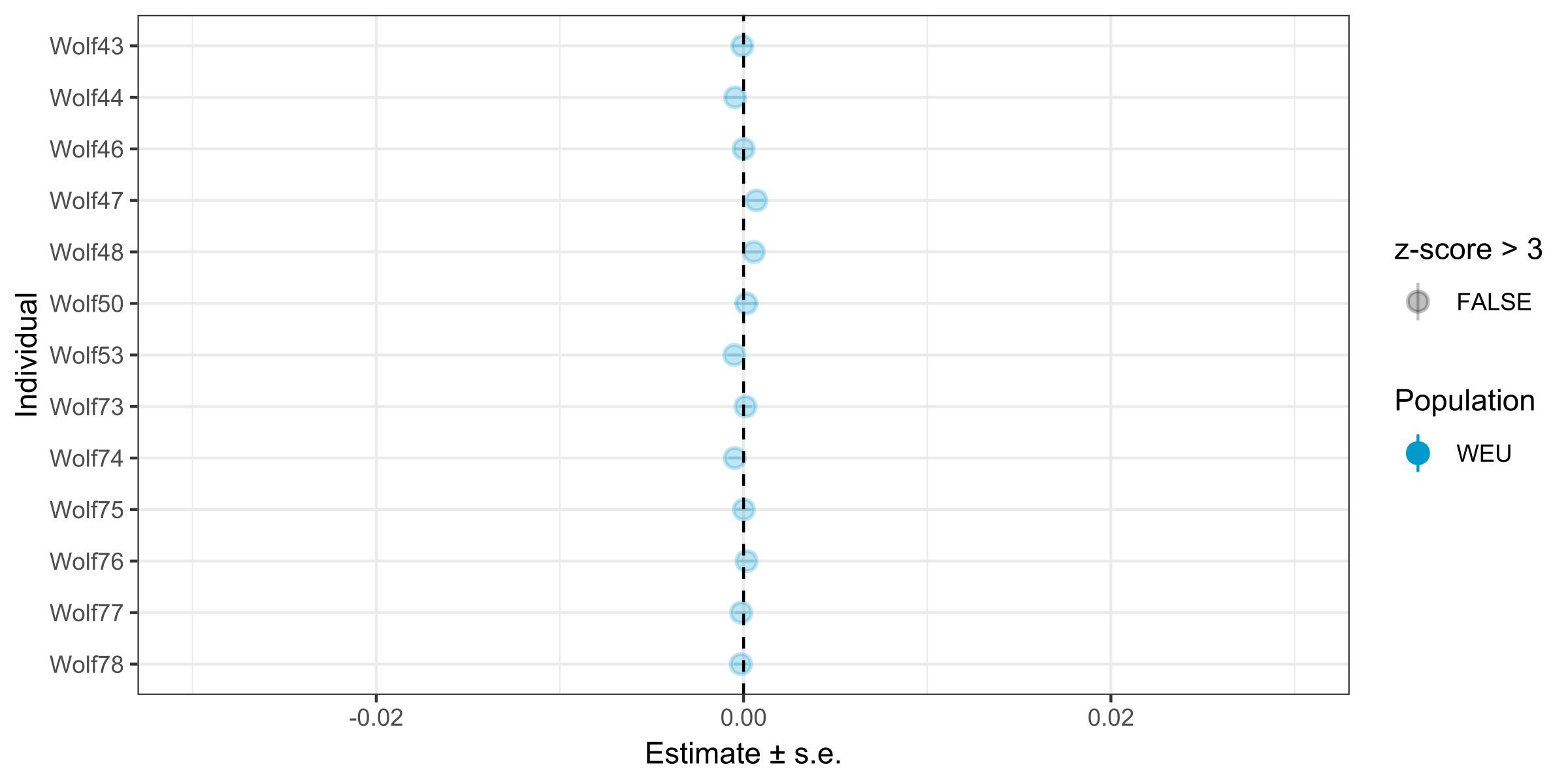

Supplement: Supplementary file 8 — Figure S3: Results of the f4‐statistic analysis testing for potential gene flow between (i) each non‐admixed European wolf individual and (ii) other non‐admixed European wolves (NWIT) or (iii) dogs without Italian and European wolf ancestry (DOG), with (iv) Canis latrans used as an outgroup (OUT). Individuals with a z‐score > 3 are considered significantly introgressed genome‐wide. [file ECE3-15-e72508-s004.jpeg]

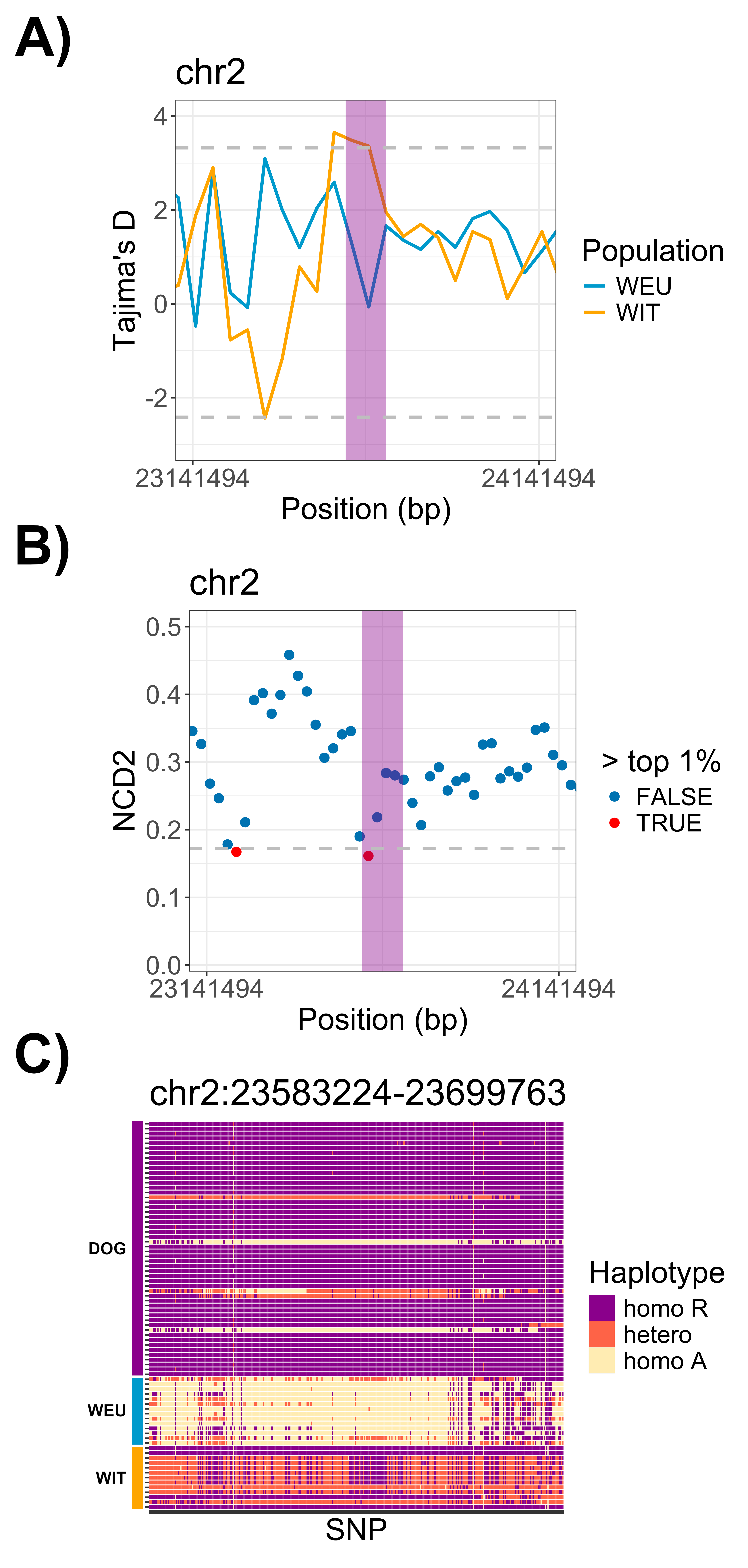

Supplement: Supplementary file 9 — Figure S4: (A) Tajima's D estimate of 1 Mb region surrounding the validated introgressed region (purple‐shaded areas) that exhibit signs of balancing selection on the entire subset of Italian wolves. The dashed gray lines represent top 1 percentiles for positive and negative Tajima's D estimates. (B) NCD2 statistics estimates of 1 Mb region surrounding the same validated introgressed region (purple‐shaded areas) that exhibit signs of balancing selection. The dashed gray lines represent top 1 percentiles. (C) Painted haplotypes for the same introgressed validated region are shown (DOG = non‐admixed dogs; WIT = admixed & non‐admixed Italian wolves sharing the region; WEU = non‐admixed European wolves). ‘R’ and ‘A’ refer to reference and alternate alleles for each SNP. [file ECE3-15-e72508-s011.jpeg]

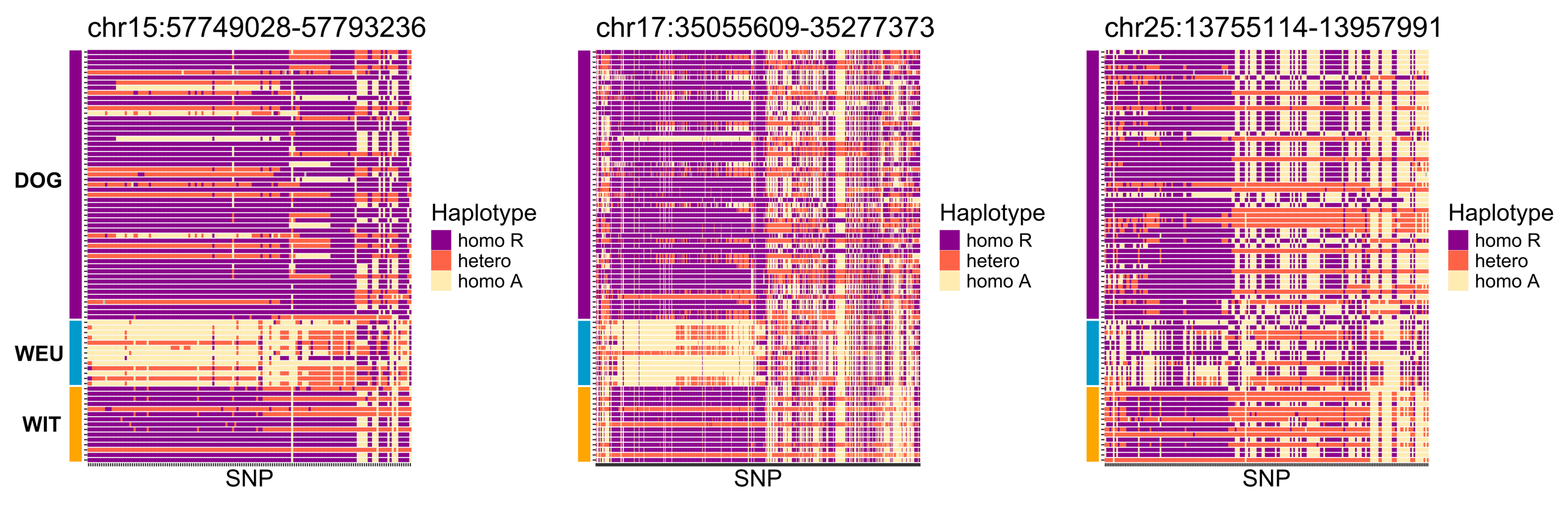

Supplement: Supplementary file 10 — Figure S5: Haplotype paintings for the three validated introgressed regions exhibiting signs of positive selection (DOG = non‐admixed dogs; WEU = non‐admixed European wolves; WIT = admixed and non‐admixed Italian wolves sharing the region). ‘R’ and ‘A’ refer to reference and alternate alleles for each SNP. [file ECE3-15-e72508-s010.jpeg]

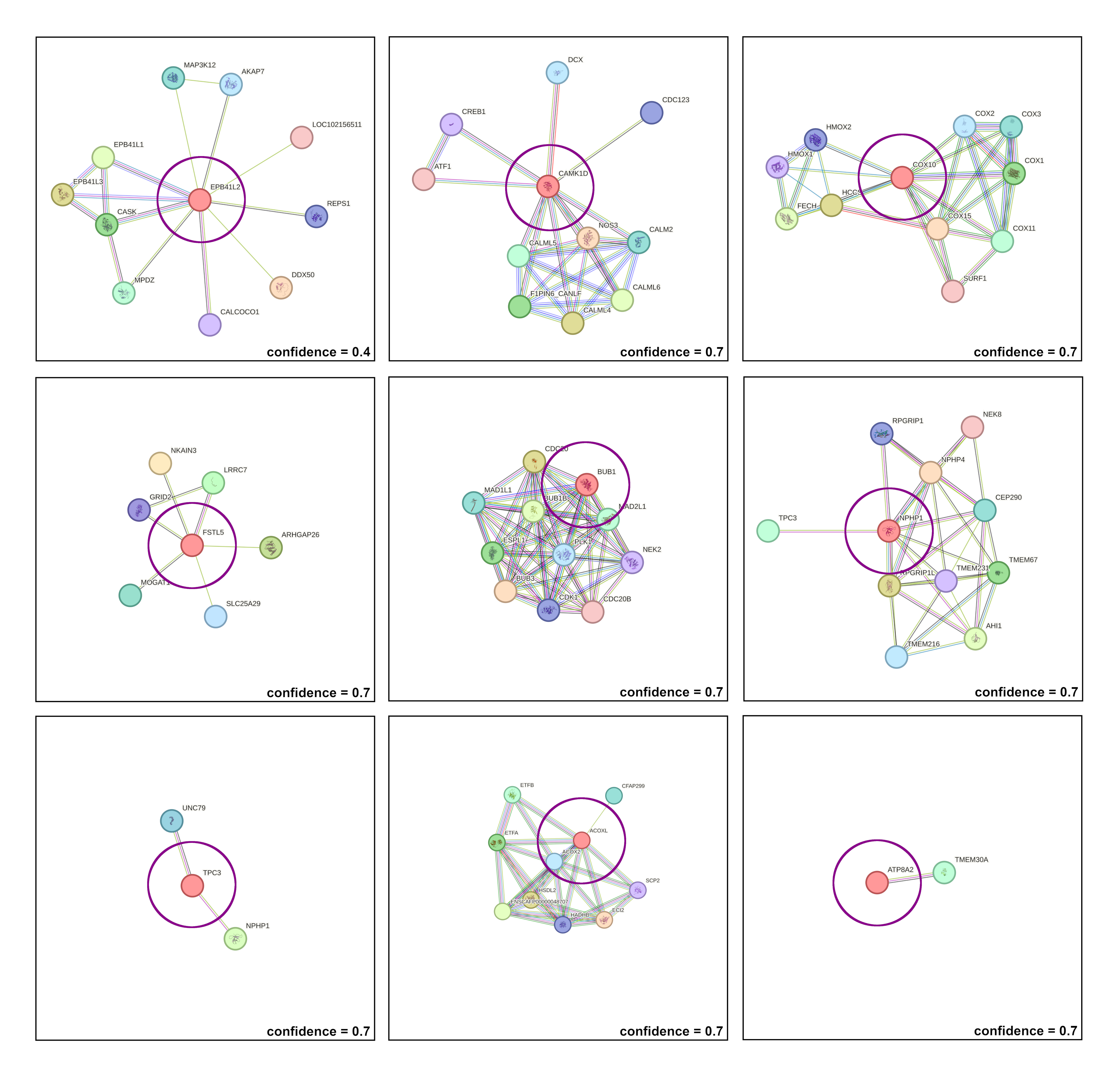

Supplement: Supplementary file 11 — Figure S6: Significant gene networks obtained with STRING for the genes on the validated introgressed regions on the top 10th percentile of non‐admixed & admixed Italian wolves (WIT) shared regions. Each network confidence is specified. The purple circle surrounds the candidate gene. [file ECE3-15-e72508-s009.jpeg]
