# Supplementary material for: Whole‐Genome Data to Investigate Recent and Historical Dog Introgression Patterns in Italian Wolves
Source: Ecol Evol. 2025 Nov 27;15(12):e72508. doi: 10.1002/ece3.72508 (PMC12658625; doi:10.1002/ece3.72508)
Supplement: Supplementary file 12 — Supinfo S1. ece372508‐sup‐0012‐SupinfoS1.docx. [file ECE3-15-e72508-s006.docx]

*Supplementary Table 1* – Whole-genome dataset. Yellow = related individual removed from the dataset; orange = non-admixed Italian wolf; red = admixed Italian wolf; blue = non-admixed European wolf; purple = non-admixed dog; green = outgroup.

*Supplementary Table 2* – Genotype filtering summary for each kind of analyses.

*Supplementary Table 3* – List of genes associated with behavioral traits in dogs and captive wolves, tested using the ‘Behavior-related genes assessment and shuffling’ approach.

*Supplementary Fig. 1* – A) Cross-validation errors estimated for each number of cluster (K) in the first ADMIXTURE run. B) ADMIXTURE plots based on the whole dataset, with K=2, K=5, and K=10, representing dogs (DOG), Italian wolves (WIT), European wolves (WEU), Asian wolves (WASIA), and North American wolves (WNA).

*Supplementary Fig. 2* – Most likely admixture pedigree (Pedigree 1) compared to the indipendent ancestries pedigree for each recently admixed individual as a result of ‘apoh’ analyses.

*Supplementary Table 4* – 'apoh' output for each WIT individual. Admixture pedigree compatibility is measured as the 'distance' between the paired ancestries expected under the 'indipendent' pedigree and the estimated ones. If pedigree 1 or 2 exhibits a lower value compared to the 'indipendent' one than the individual is recently admixed (colored in red). We also highlighted one individual non-recently admixed but with close distances (in yellow).

*Supplementary Fig. 3* – Results of the f4​-statistic analysis testing for potential gene flow between (i) each non-admixed European wolf individual and (ii) other non-admixed European wolves (NWIT) or (iii) dogs without Italian and European wolf ancestry (DOG), with (iv) Canis latrans used as an outgroup (OUT). Individuals with a z-score > 3 are considered significantly introgressed genome-wide.

*Supplementary Fig. 4* – A) Tajima’s D estimate of 1 Mb region surrounding the validated introgressed region (purple-shaded areas) that exhibit signs of balancing selection on the entire subset of Italian wolves. The dashed grey lines represent top 1 percentiles for positive and negative Tajima’s D estimates. B) NCD2 statistics estimates of 1 Mb region surrounding the same validated introgressed region (purple-shaded areas) that exhibit signs of balancing selection. The dashed grey lines represent top 1 percentiles. C) Painted haplotypes for the same introgressed validated region are shown (DOG = non-admixed dogs; WIT = admixed & non-admixed Italian wolves sharing the region; WEU = non-admixed European wolves). ‘R’ and ‘A’ refer to reference and alternate alleles for each SNP.

*Supplementary Fig. 5* – Haplotype paintings for the three validated introgressed regions exhibiting signs of positive selection (DOG = non-admixed dogs; WEU = non-admixed European wolves; WIT = admixed and non-admixed Italian wolves sharing the region). ‘R’ and ‘A’ refer to reference and alternate alleles for each SNP.

*Supplementary Table 5* – Gene ontology network results using STRING on genes within introgressed and validated regions in admixed Italian wolves (i.e., gene PREX2), and all the Italian wolves (the other genes). Green = functional enrichment for the candidate gene.

*Supplementary Fig. 6* – Significant gene networks obtained with STRING for the genes on the validated introgressed regions on the top 10^th^ percentile of non-admixed & admixed Italian wolves (WIT) shared regions. Each network confidence is specified. The purple circle surrounds the candidate gene.
